# Imaging diagnosis of autoimmune pancreatitis: computed tomography and magnetic resonance imaging

**DOI:** 10.1007/s10396-021-01145-8

**Published:** 2021-10-26

**Authors:** Hiroshi Ogawa, Yasuo Takehara, Shinji Naganawa

**Affiliations:** 1 Department of Radiology, Nagoya University Graduate School of Medicine, 65 Tsurumai, Showa, Nagoya 466-8550 Japan; 2 Department of Fundamental Development for Advanced Low Invasive Diagnostic Imaging, Nagoya University Graduate School of Medicine, 65 Tsurumai, Showa, Nagoya 466-8550 Japan

**Keywords:** IgG4, Autoimmune pancreatitis, Computed tomography, Diffusion-weighted image, MR elastography

## Abstract

Autoimmune pancreatitis (AIP) is a pancreatic phenotype of IgG4-related systemic disease. Since its first description in the literature, characteristic imaging features have gradually become known to many clinicians encompassing various specialties in the past quarter century. CT and MRI have been the workhorses for imaging diagnosis of AIP. Typical features include sausage-like swelling of the focal or entire pancreas, duct-penetrating sign, a capsule-like rim of the affected lesions, and homogeneous delayed enhancement or enhanced duct sign after contrast administration, as well as characteristic combined findings reflecting coexisting pathologies in the other organs as a systemic disease. In this review, recent and future developments in CT and MRI that may help diagnose AIP are discussed, including restricted diffusion and perfusion and increased elasticity measured using MR.

## Introduction

Autoimmune pancreatitis (AIP) was first reported by Yoshida et al. [1] in 1995 as a disease that presents diffuse pancreatic swelling and tapered pancreatic ductal stenosis, ameliorated by steroid administration. Since then, AIP has been understood as a part of systemic IgG4-related diseases. The diagnostic criteria for AIP include the International Consensus Diagnostic Criteria (ICDC) [2] internationally, and the Japanese Clinical Diagnostic Criteria [3] in Japan. In these diagnostic criteria, pancreatic swelling (diffuse or segmental/focal) and irregular stenosis of the main pancreatic duct are described as items for diagnostic imaging. As data from more cases have become available, characteristic imaging findings on various modalities are gradually becoming known to many clinicians encompassing various specialties. In this article, representative findings of AIP on computed tomography (CT) and magnetic resonance imaging (MRI), which are considered the most versatile, and the results of recent research and their future potential, are shared.

## CT and MRI findings of AIP

Contrast-enhanced CT has been the workhorse in the diagnosis of AIP. The iodinated contrast medium used for CT is a non-specific extravascular contrast medium and thereby is distributed into the fibrosis induced by AIP. The gadolinium chelate for MRI behaves similarly to the iodinated contrast medium, enhancing the fibrotic component of the tissues affected by AIP.

MRI is also a powerful tool in AIP diagnosis because of its inherent tissue-characteristic contrasts. For instance, normal pancreatic tissue is depicted by a clear high signal on fat-saturated T1-weighted images; however, the high signals are lost in the area affected by AIP. Similarly, fibrosis is characterized by low intensity on T2-weighted images; therefore, the capsule-like rim demarcates the swollen pancreas with a low signal. In addition, a high signal on the diffusion-weighted image represents highly cellular plasmacyte proliferations. In addition, MRI can add physical properties such as tissue stiffness or rigidity into imaging contrast. Highly fibrotic tissues are associated with tissue stiffness; therefore, MRI can depict fibrotic AIP lesions on MR elastography [4].

### Pancreatic enlargement (diffuse or localized)

**Fig. 1:**
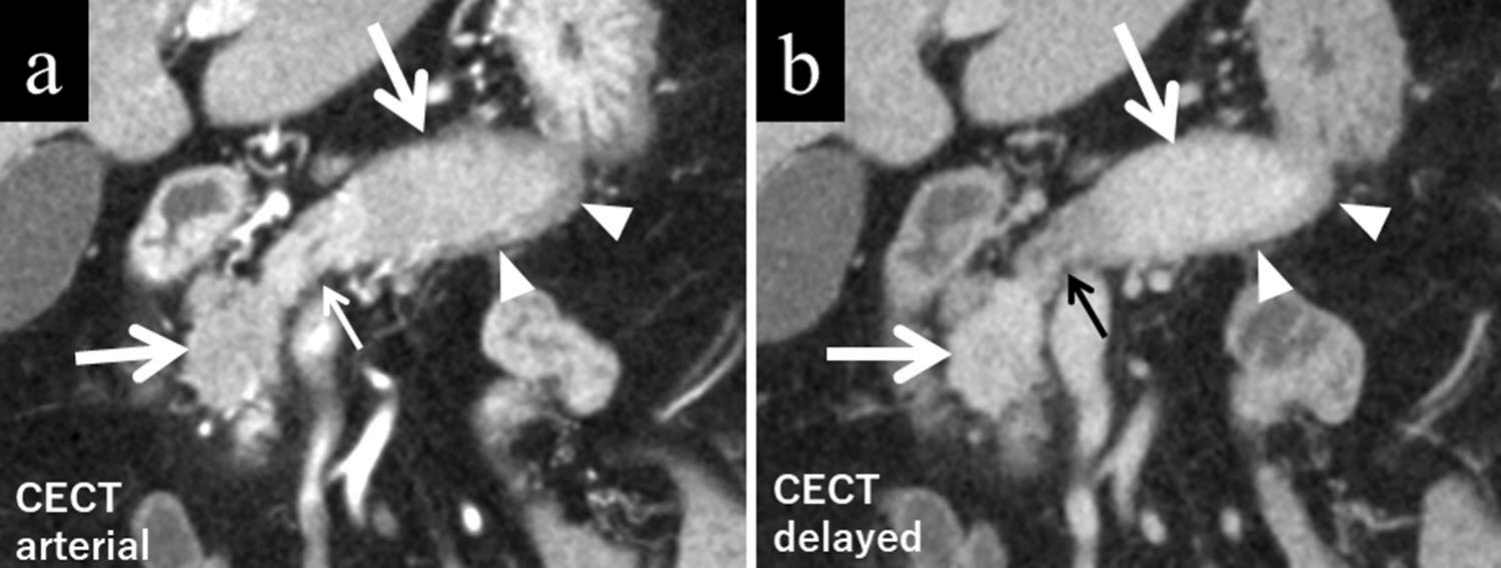
A male patient in his 70 s with typical autoimmune pancreatitis (AIP). **a** There is localized sausage-like swelling of the pancreas affected by AIP (large arrows). These lesions are hypodense during the pancreatic phase as compared to the normal pancreas (small arrow). Typical rim enhancement (arrowheads) demarcating the pancreatic tail is seen. **b** The same coronal section on delayed-phase CT reveals the lesion is hyperdense during the delayed phase compared to the normal pancreatic parenchyma (small arrow). The rim is also discernible in the delayed phase (arrowheads)

**Fig. 2:**
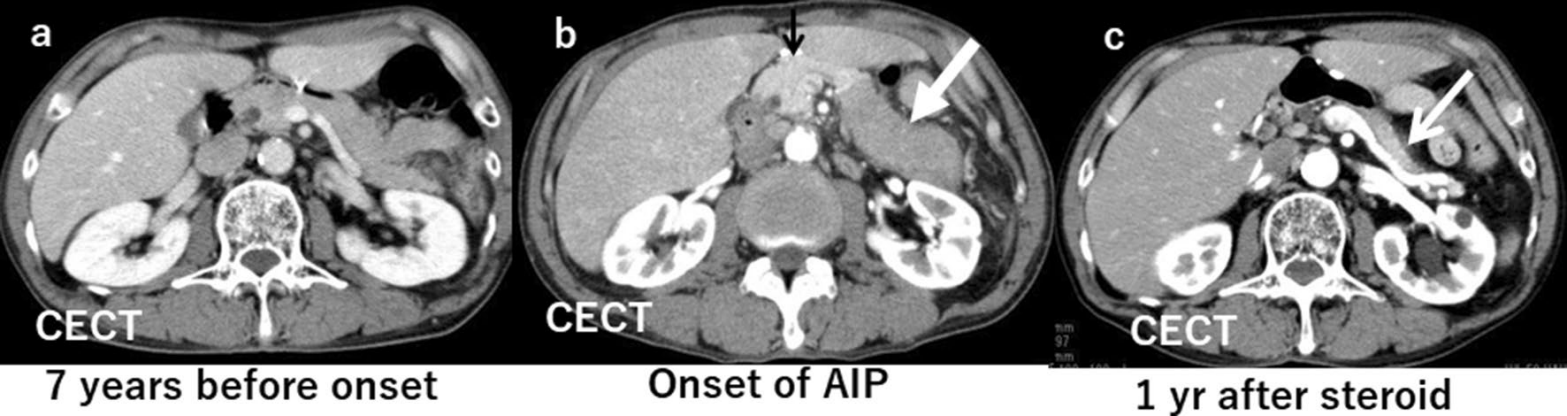
A male patient in his 70 s with autoimmune pancreatitis (AIP). **a** Seven years before the onset of AIP, there was no pancreatic swelling on contrast-enhanced CT (CECT). **b** At the onset of AIP, there is distinct sausage-like swelling of the pancreatic body to tail on CECT. The area affected by AIP is a homogeneously less enhanced area (large arrow) as compared to the unaffected area (small arrow). **c** One year after initiation of prednisolone, the affected lesion returned to a normal size and enhancement on CECT (arrow)

The findings of AIP are created by significant lymphocytes or plasma cell infiltration and fibrosis. The appearance is often compared to sausage because the typical lobular structure disappears, and the margin of the pancreas becomes straight (Fig. 1a, b). The definition of pancreatic swelling has classically been dependent on the criteria proposed by Haaga et al. [5]. Still, in reality, it is better to consider the age and background of the patient comprehensively. Since the pancreas decreases in volume with age, a disproportionately large pancreas in an older patient suggests an abnormality. In such instances, it is strongly recommended to refer to previous images (Fig. 2a–c).

### Homogeneously decreased enhancement in the arterial or pancreatic phase

Unlike the liver with its dual blood supplies, the pancreas is perfused solely by arterial blood flow; therefore, the healthy pancreas is characterized by a steep and homogeneous rise of enhancement after intravenous contrast administration. The area affected by AIP shows reduced enhancement in the arterial or pancreatic phase of contrast-enhanced dynamic CT (Fig. 1a).

### Homogeneous delayed enhancement

In contrast to the reduced enhancement in the pancreatic phase, the affected area shows increased enhancement in the delayed phase compared to the normal pancreatic parenchyma (Fig. 1b). The contrast enhancement in the delayed phase is uniform [6], reflecting acinar cell shedding and a high degree of fibrosis. Homogeneous delayed enhancement is a useful finding for differentiation of AIP from pancreatic ductal adenocarcinoma (PDAC) because its sensitivity, specificity, and accuracy are reported to be 59–100%, 65–94.9%, and 68.3–94.3%, respectively [6–13].

### Punctate enhancements in the pancreatic phase within the lesion

**Fig. 3:**
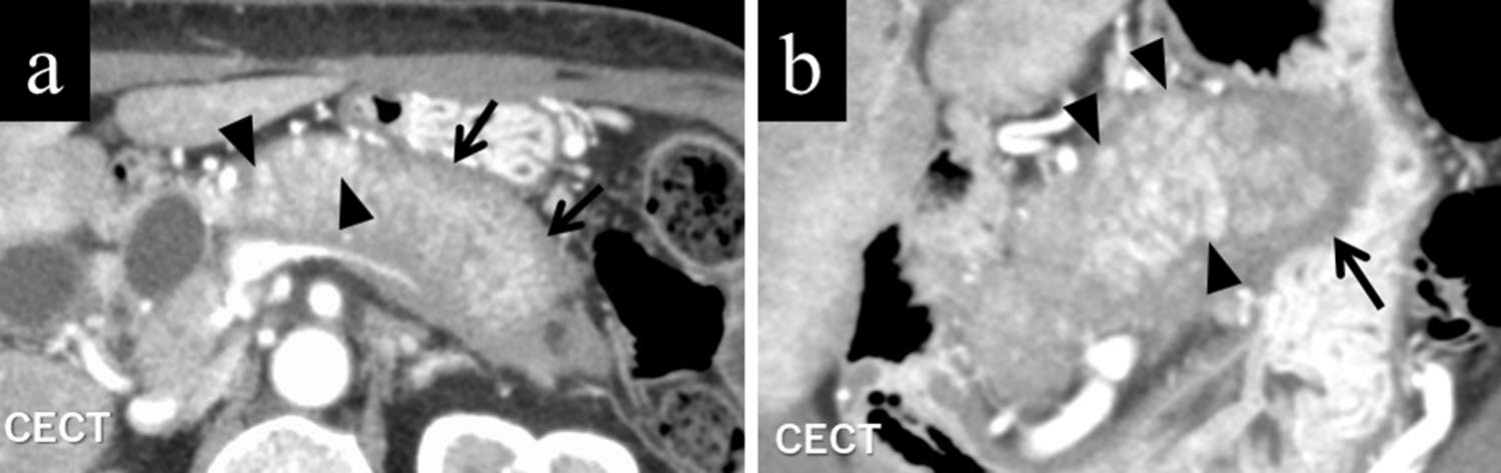
A female in her 60 s with typical autoimmune pancreatitis. **a** Axial contrast-enhanced CT in pancreatic phase. The pancreas is swollen as a whole, and punctate contrast-enhanced areas inside (arrowheads), as well as a typical band-shaped structure demarcating the lesion (arrows), are shown. **b** Coronal reconstruction of the same phase. Punctate contrast-enhanced areas (arrowheads) and a band-shaped structure surrounding the lesion (arrows) are also shown on the coronal image

The normal pancreatic parenchyma may focally remain within the diffusely affected AIP lesions. Such areas may still hold inherent arterial blood perfusion and thereby be depicted as punctate, speckled, or dotted contrast enhancements [7] (Fig. 3a, b). Punctate enhancements in the pancreatic phase within the lesion are useful findings for differentiation of AIP from PDAC, with its sensitivity, specificity, and accuracy reported to be 50–88.9%, 48.6–95%, and 62–91.7%, respectively [7, 10, 13].

### Enhanced duct sign

**Fig. 4:**
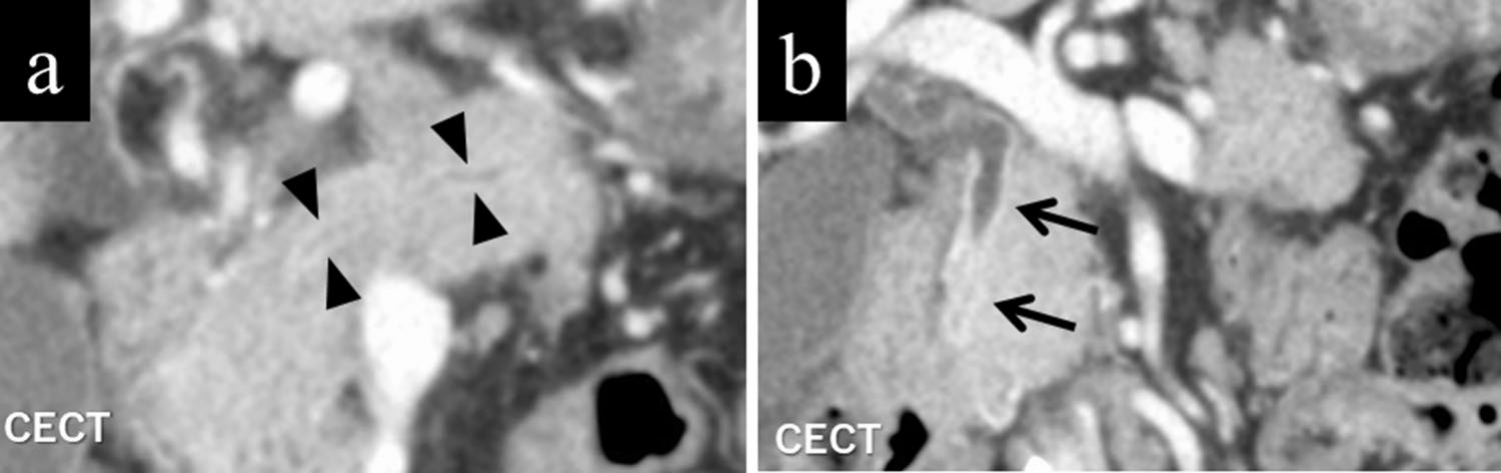
Coronal reconstruction images of contrast-enhanced portal-venous phase CT of a male in his 70 s with typical autoimmune pancreatitis. **a** Contrast-enhanced segments demarcating the wall of the main pancreatic duct (enhanced duct sign) are observed (arrowhead). **b** The enhancement demarcating the common bile duct wall is also evident (arrows), reflecting coexisting sclerosing cholangitis

In AIP, a contrast-enhanced area along the MPD wall may be seen [10, 14, 15] (Fig. 4a), which is thought to reflect the inflammations around the pancreatic duct. Furuhashi et al. [10] reported that the enhanced duct sign was a useful finding for differentiation of AIP from PDAC, with a sensitivity, specificity, and accuracy of 36%, 98%, and 82%, respectively.

### Capsule-like rim

**Fig. 5:**
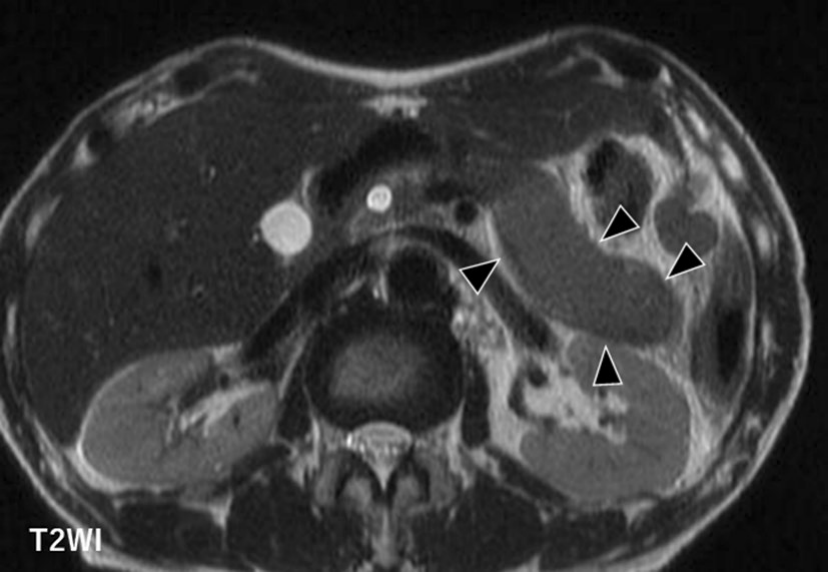
A male patient in his 70 s. On T2-weighted image, autoimmune pancreatitis-affected area shows slightly high intensity demarcated by a low-intensity rim

A band-like rim structure may be found in whole or in part demarcating the pancreas affected by AIP [14] (Figs. 1a, b, 3a, b). This finding is believed to reflect a high degree of fibrosis. CT shows an AIP-affected area as low density before contrast administration, whereas gradual contrast enhancement follows in the dynamic study. MRI shows a corresponding low signal rim on T2-weighted images reflecting these fibrotic areas (Fig. 5). Capsule-like rim is a useful finding for differentiation of AIP from PDAC because its sensitivity, specificity, and accuracy are reported to be 10–64%, 90.9–100%, and 70–93%, respectively [7–10, 12, 13, 16–18].

**Fig. 6:**
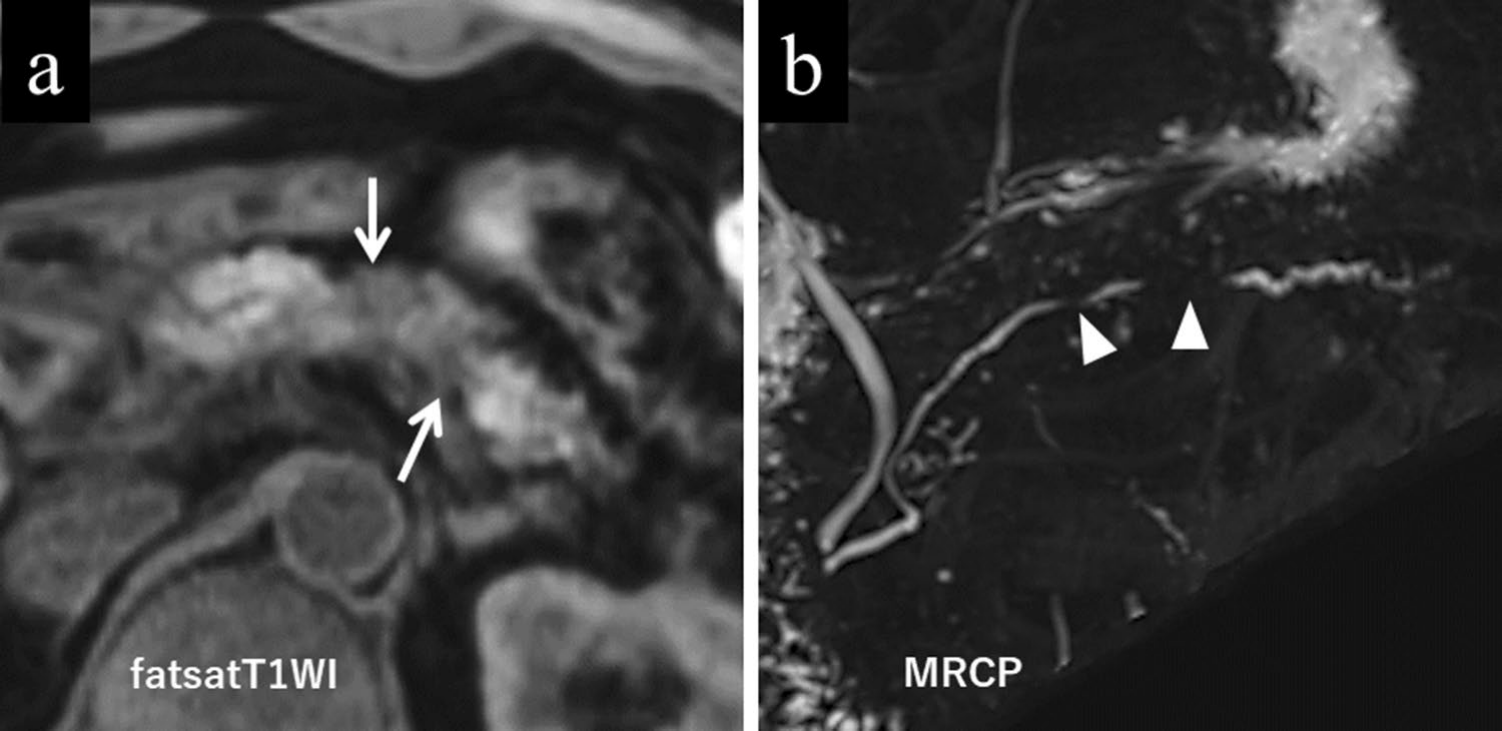
Typical MRI images seen in a male patient in his 50 s with autoimmune pancreatitis. **a** The lesion is hypointense on fat-saturated T1-weighted image (arrows). **b** Multiple stenotic segments of the main pancreatic duct (skip narrowing and the icicle sign) are seen (arrowheads) on MRCP

The contrast changes seen in AIP on MRI are more characteristic, if not specific, than on CT. On MRI fat-saturated T1-weighted images, normal pancreatic tissue is inherently characterized by a high signal; however, the integrities of the signal intensity and the texture of the pancreatic exocrine glands are lost in the affected lesion (Fig. 6a).

### Duct-penetrating sign

In AIP, the main pancreatic duct (MPD) may penetrate the lesion without complete occlusion. In addition, the MPD stenosis in the lesion may taper, which is called the icicle sign [18]. Multiple skip narrowings of the MPD may also be seen (Fig. 6b) [17]. Magnetic resonance cholangiopancreatography (MRCP), its depiction not depending on luminal contrast delivery upstream to the stenosis, is suitable for depicting the entire MPD pathologies affected by AIP. Therefore, MRCP can readily display the icicle sign in the multiple skipped segments. The duct-penetrating sign is a useful finding for differentiation of AIP from PDAC, with its sensitivity, specificity, and accuracy reported to be 13.3–73%, 91.4–100%, and 68–93%, respectively [7, 8, 10, 11, 13, 17, 18].

### Imaging findings of AIP after steroid therapy

In cases of AIP, steroid treatment improves pancreatic swelling [19, 20]. In some cases, the pancreatic parenchyma may be more atrophic than before treatment. The capsule-like rim disappears and the main pancreatic duct stenosis improves. In our experience, the enhanced duct sign also disappears, but the contrast enhancement of the pancreatic parenchyma may not be completely normalized. At the time of recurrence, these abnormal findings will reappear.

## Extrapancreatic lesions

Typical extrapancreatic lesions of AIP include sclerosing cholangitis [21] (Fig. 4b), retroperitoneal fibrosis [22], and interstitial nephritis [23]. In sclerosing cholangitis, the bile duct wall is thickened, and the contrast enhancement is increased. Retroperitoneal fibrosis is often visualized on CT as a soft tissue density area surrounding the abdominal aorta or bilateral common iliac arteries. Lesions of interstitial nephritis are seen as areas of poor contrast enhancement and are visualized as low-intensity lesions on T2-weighted images. In addition, lacrimal gland or salivary gland inflammation [24], interstitial pneumonia [25], and lymphadenopathy [26] may be seen. These findings are not always present; however, it is a basis for suspicion of AIP if found.

## Recent research

### Diffusion-weighted images (DWI)

**Fig. 7:**
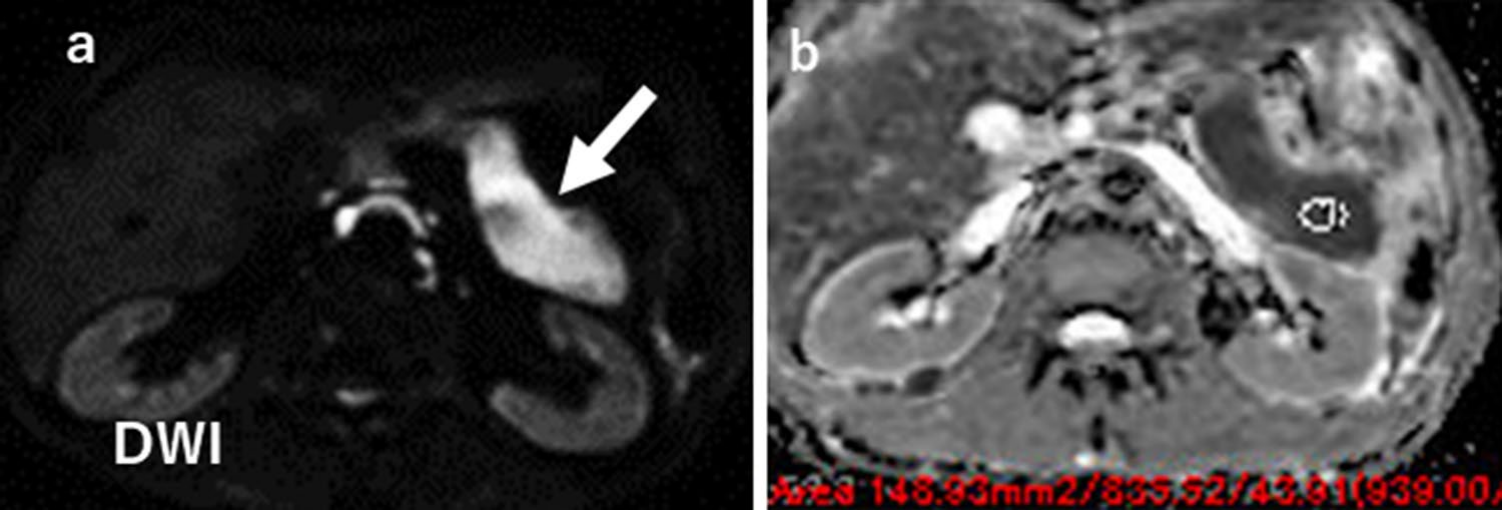
A male patient in his 70. **a** On diffusion-weighted image, a homogeneously hyperintense body to tail (arrow) suggests highly cellular plasmacyte proliferation seen in autoimmune pancreatitis. **b** On the ADC map, the ADC value of the lesion is 0.8 × 10^–3^ mm^2^/s

Due to plasma cell proliferation, the AIP lesion shows a high signal on diffusion-weighted images (DWI) (Fig. 7a). Ren et al. reported that the apparent diffusion coefficient (ADC) map helped distinguish mass-forming-type AIP from PDAC [27]. They found that the areas under the curve (AUCs) of receiver operating characteristic (ROC) were maximized when using the maximum ADC value as a parameter. Choi et al. reported that the mean ADC value of the lesion was significantly lower in mass-forming AIP than in PDAC [11]. The sensitivity and specificity were 66.7% and 81.0%, respectively, when the ADC values were separated by 0.9407 × 10^–3^ mm^2^/s (Fig. 7b). Zhu et al. investigated mean ADC values after treatment with AIP and recurrence [28]. According to them, the ADC value of AIP is increased by steroid treatment, but not so much at the time of recurrence. Therefore, they reported that the accuracy might decrease when predicting recurrence using the ADC value. Sekito et al. investigated the usefulness of ADC values in differentiating type 1 localized AIP from PDAC and further determining the therapeutic effect. They reported that the mean ADC values of the lesions were significantly lower in AIP than in PDAC and were elevated considerably after steroid treatment [29].

### Intravoxel incoherent motion (IVIM)

Klauss et al. compared perfusion rates of AIP, PDAC, and the normal pancreas in terms of intravoxel incoherent motion (IVIM) [30]. They reported that the perfusion rate was significantly lower in AIP than in the normal pancreas, and in addition, the rate was the lowest in PDAC. Interestingly, the rate of AIP perfusion increased after steroid therapy.

### MR elastography (MRE)

**Fig. 8:**
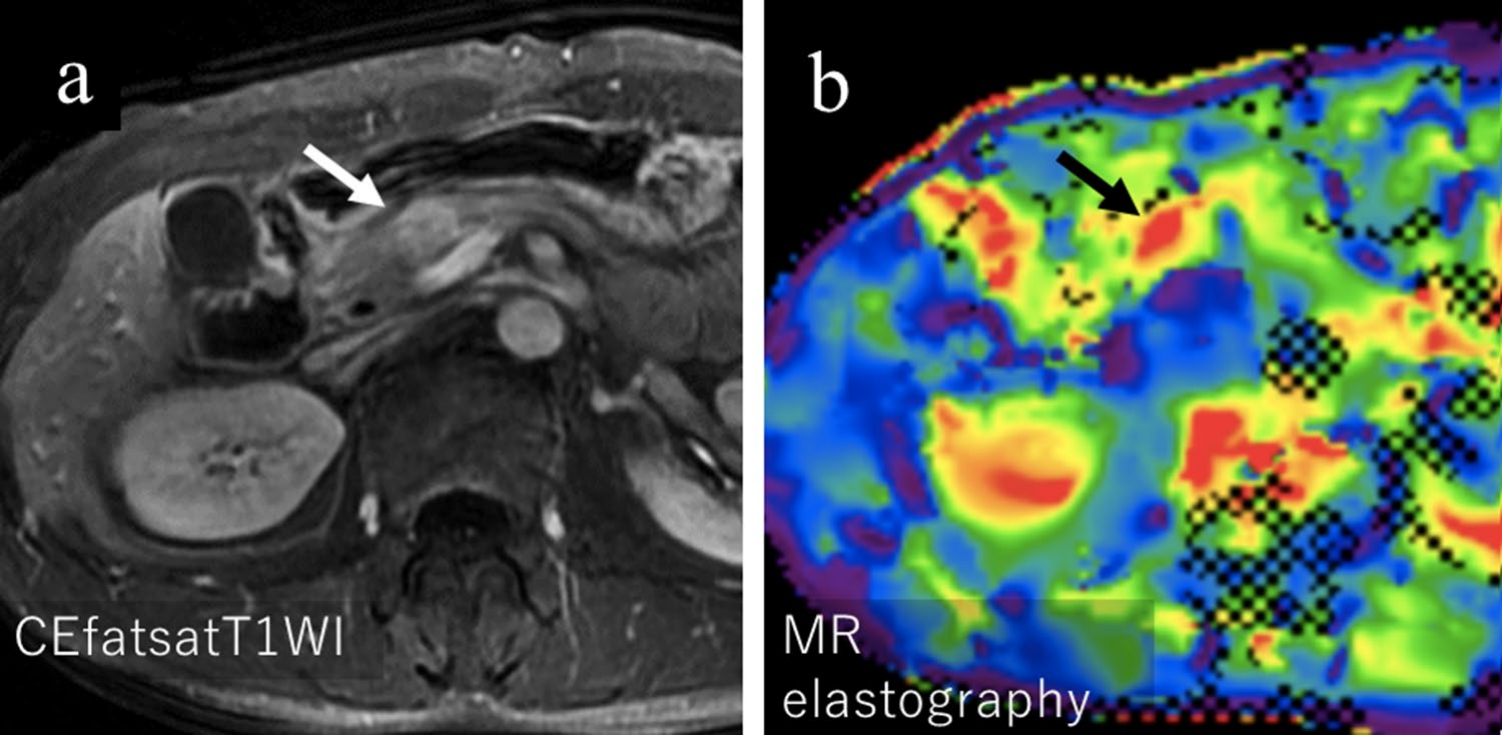
A male in his 50 s with IgG4-related autoimmune pancreatitis (AIP) associated with pathologically proven desmoplastic inflammatory pseudotumor. **a** Contrast-enhanced T1-weighted image showing delayed positive enhancement of the lesion in the pancreatic head (arrow). **b** A color-coded stiffness map shows a high stiffness of 6.2 kPa in the lesion (arrow), higher than previously reported values for AIP

Shi et al. compared the rigidity of AIP with that of PDAC using MR elastography (MRE). They reported the rigidity or stiffness of PDAC and AIP [31] concluding the median value was significantly lower in AIP (2.67 kPa [interquartile range 2.24–3.56 kPa]) than in PDAC (3.78 kPa [3.22–5.11 kPa]); however, in our experiences, the stiffness of AIP considerably varies depending on its pathological phases (Fig. 8a, b).

Besides MRI, ultrasound elastography may be promising since similar information concerning the pancreas stiffness can be obtained with better spatial resolution than MRE [32–35].

## Conclusions

Imaging diagnosis of AIP is not very difficult thanks to its characteristic findings. CT and MRI are the most standard methods for the diagnosis, and further cutting-edge research and developments are underway.
